# Rab25 expression predicts poor prognosis in clear cell renal cell carcinoma

**DOI:** 10.3892/etm.2014.1867

**Published:** 2014-07-29

**Authors:** LUNZHI LIU, GUOHUA DING

**Affiliations:** Division of Nephrology, Renmin Hospital of Wuhan University, Wuhan, Hubei 430060, P.R. China

**Keywords:** clear cell renal cell carcinoma, Rab25, prognosis, marker

## Abstract

Rab25 has been implicated in a number of types of cancer. However, its expression status and clinical implications in clear cell renal cell carcinoma (ccRCC) remain to be investigated. The purpose of this study was to investigate the significance of Rab25 status in patients with ccRCC. Rab25 expression was determined by western blot analysis in 30 fresh ccRCC samples. Immunohistochemistry was performed on the ccRCC samples and paired adjacent noncancerous tissues from 107 patients with ccRCC who had undergone surgery. The prognostic role and correlations with other clinicopathological factors were evaluated. Rab25 expression was upregulated in ccRCC tissues compared with that in paired adjacent noncancerous tissues. A high expression of Rab25 protein was significantly correlated with the primary tumor stage; lymph node metastasis; distant metastasis; tumor, node and metastasis stage and histological grade. A Kaplan-Meier survival analysis by log-rank test demonstrated that elevated Rab25 expression predicted lower overall survival time in patients with ccRCC. Notably, multivariate analyses revealed that expression of Rab25 was an independent prognostic factor in ccRCC (hazard ratio, 3.43; 95% confidence interval, 1.13–10.38; P=0.023). In conclusion, Rab25 is a potential prognostic biomarker in ccRCC.

## Introduction

Renal cell carcinoma (RCC) is the most lethal common urologic cancer, accounting for 3% of all types of cancer in adults (1). Surgical resection is the principal type of treatment. However, >40% of patients with RCC develop metastases following radical nephrectomy, and the 10-year cancer-specific survival rate is poor (2). Clear cell RCC (ccRCC) is the most common type of RCC occurring in adults, and is associated with a worse prognosis than the other two major subtypes, chromophobe and papillary RCC (3,4). The five-year disease-specific survival rate of ccRCC is <70% (5,6). Biomarkers that could identify the aggressive potential of ccRCC may shape appropriate therapeutic strategies earlier in the course of the malignancy. Therefore, a number of groups studying ccRCC have focused on identifying promising molecular markers that are associated with the progression of ccRCC.

Rab25 is a member of the Rab family of small guanosine triphosphatase (GTPase) proteins; these proteins are considered key regulators of intracellular membrane trafficking involved in multiple diseases, including cancer (7,8). Previous studies have indicated that Rab25 functions as a tumor suppressor or as an oncogene, depending on the tumor type. The downregulation of RAB25 gene expression has been shown to correlate with tumor progression and poor prognosis in colon, esophageal and breast cancer (9–11). By contrast, the overexpression of RAB25 has been associated with decreased survival rate and a more aggressive disease phenotype in ovarian and bladder cancer (12,13). However, the expression pattern and clinical implications of Rab25 protein in ccRCC have not been studied to date. In this study, the association between Rab25 expression and clinical outcome in patients with ccRCC was analyzed.

## Materials and methods

### Patients and tissue specimens

Specimens of ccRCC and paired noncancerous renal tissues (n=107) were collected from patients in the Division of Nephrology, Renmin Hospital of Wuhan University (Wuhan, China) between January 2006 and December 2011. All patients were newly diagnosed with ccRCC by histopathology and none had received any previous treatment. Noncancerous control renal tissues were surgically excised from the same patients. All specimens were obtained immediately subsequent to surgical removal, snap-frozen in liquid nitrogen and stored at −80°C. Informed consent was obtained from all patients. Information on the recurrence or metastasis of ccRCC and disease-related mortality was obtained based on the review of medical records. Tumor stage and nuclear grading were classified according to the 2010 tumor, node and metastasis (TNM) staging system (14). This study was approved by the Institutional Review Board of Renmin Hospital of Wuhan University.

### Western blotting

ccRCC tissues were resolved using a 10% polyacrylamide gel in a sodium dodecyl sulfate buffer by electrophoresis. Subsequent to being transferred onto a nitrocellulose membrane, the blots were incubated with anti-Rab25 antibody (Abcam, Cambridge, MA, USA). Binding of Rab25 antibody was revealed by chemiluminescence following incubation with horseradish peroxidase (HRP)-conjugated goat anti-mouse antibody (Bio-Rad Laboratories, Hercules, CA, USA). GAPDH (Cell Signaling Technology, Inc., Danvers, MA, USA) was used as the internal control.

### Immunohistochemical detection of Rab25

Paraffin sections were deparaffinized in xylene and rehydrated in graded alcohols and distilled water. Slides were heated for antigen retrieval in 10 mmol/l citrate (pH 6.0). Sections were incubated with polyclonal rabbit anti-human Rab25 antibody (1:50; Abcam) overnight at 4°C. EnVision^®^ + System-HRP (3,3′-diaminobenzidine) (Dako, Carpinteria, CA, USA) was used according to the manufacturer’s instructions. Staining was revealed by counterstaining with hematoxylin. The evaluation of immunohistochemical staining for Rab25 was conducted by two independent pathologists who had no knowledge of patient data.

### Statistical analysis

One-way analysis of variance, followed by the least significant difference post hoc test was used to compare the mean differences among different groups. Associations between categorical variables were evaluated using the Pearson’s χ^2^ test. P<0.05 was considered to indicate a statistically significant difference. These statistically significant variables were found in the univariate and multivariate analyses. All statistical analysis was performed using SPSS version 16.0 software (SPSS, Inc., Chicago, IL, USA).

## Results

### Upregulated Rab25 expression in ccRCC

**Figure 1:**
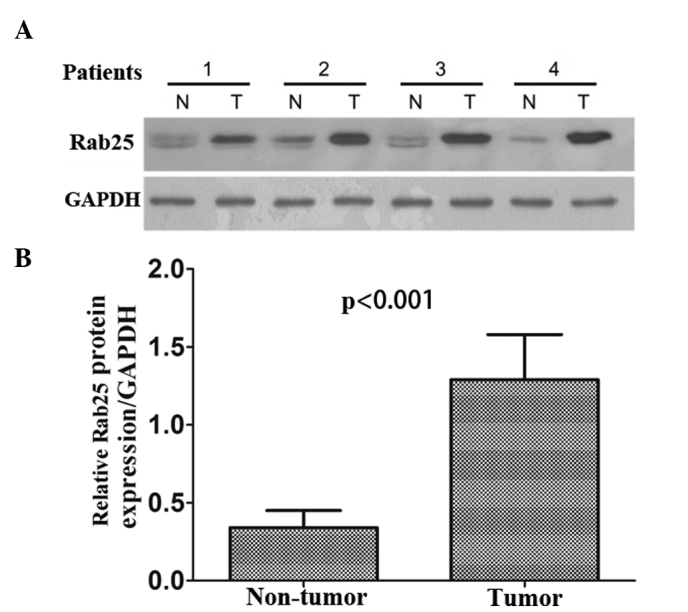
Increased Rab25 protein expression in ccRCC. (A) Representative western blots of Rab25 expression in four ccRCC and paired adjacent noncancerous tissue samples. (B) Semi-quantitative analysis of Rab25 protein expression levels relative to GAPDH in 30 fresh samples. N, non-tumor tissue; T, tumor tissue; ccRCC, clear cell renal cell carcinoma.

The Rab25 protein expression among 30 fresh ccRCC specimens and paired adjacent noncancerous tissues was evaluated. The expression of Rab25 at the protein level was significantly increased in the ccRCC tissues in comparison with that in the adjacent non-carcinoma tissues (P<0.001, Fig. 1A). Consistently, the semi-quantitative analysis of band intensity showed the same results (P<0.001, Fig. 1B).

### Rab25 expression correlates with clinicopathological features in ccRCC

**Figure 2:**
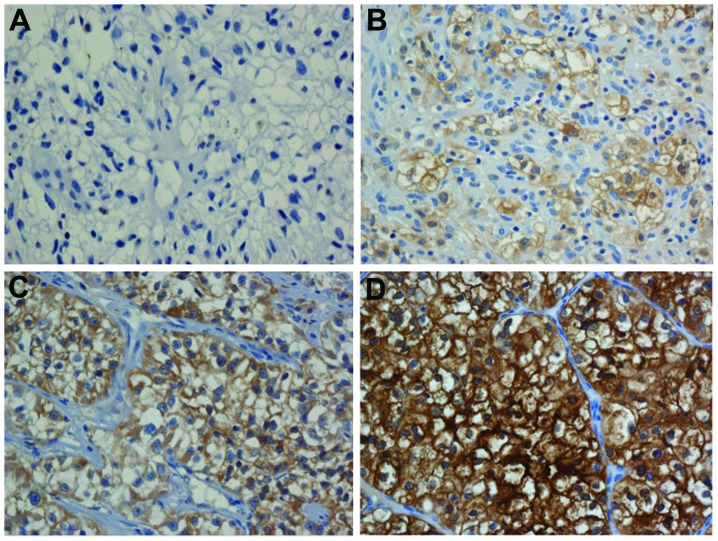
Representative example of Rab25 staining intensities. (A) 0 (no staining); (B) 1 (weak); (C) 2 (moderate); (D) 3 (strong).

An analysis of Rab25 expression was performed by immunohistochemistry for the 107 ccRCC tumor samples. The staining intensity was scored from 0 to 3 (0, no staining; 1, minimal staining; 2, moderate staining; 3, intense staining) (Fig. 2). The staining was reported to be either positive (2–3) or negative (0–1). The positive Rab25 expression was statistically higher in the ccRCC tissues than that in the paired adjacent noncancerous tissues (65.4 vs. 8.4%, P<0.001).

The correlations between Rab25 immunoreactivity and clinicopathological variables are summarized in Table I. Rab25 staining levels were significantly correlated with primary tumor stage, lymph node metastasis, distant metastasis, TNM stage and histological grade (all P<0.05).

### Rab25 expression and prognosis in patients with ccRCC

**Figure 3:**
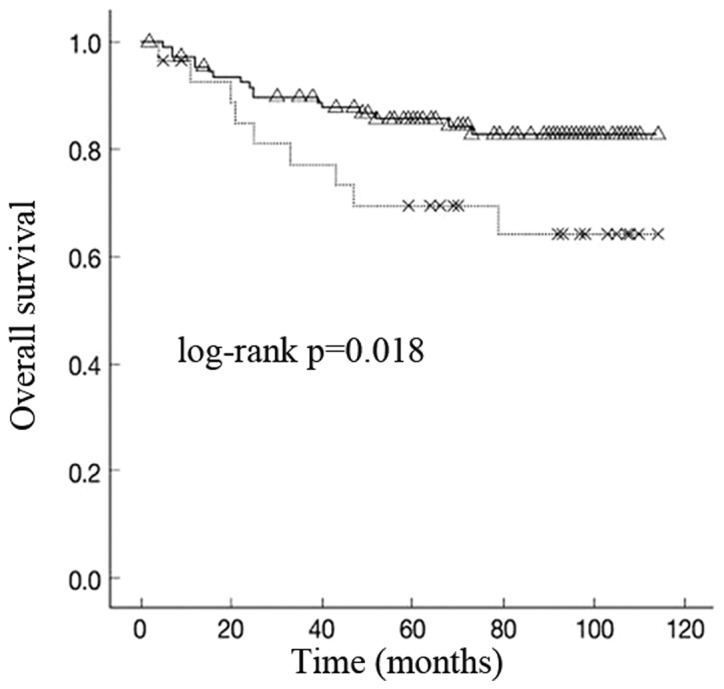
Kaplan-Meier survival analysis of overall survival rate in 107 patients with primary ccRCC with negative (n=37) and positive (n=70) Rab25 expression results following surgical resection. Log-rank test, P=0.018. ^x^Patients with positive results; ^Δ^Patients with negative results.

In a Kaplan-Meier analysis, patients with ccRCC and positive Rab25 expression had a significantly shorter post-operative overall survival time compared with those with negative Rab25 expression results (Fig. 3). To assess whether Rab25 expression was an independent predictor of overall survival time, a Cox proportional-hazards model was created, including covariates that had statistically significant correlations with overall survival time, using an inclusion threshold of P<0.05 (Table II). The univariate analysis showed that the Eastern Cooperative Oncology Group performance status, tumor size, Fuhrman’s grade, presence of vascular invasion and necrosis, and positive Rab25 status were significant predictors of poorer survival rates (all P<0.05). Furthermore, the multivariate analysis demonstrated that positive Rab25 expression remained an independent prognostic indicator for overall survival time (hazard ratio, 3.43; 95% confidence interval, 1.13–10.38; P=0.023).

## Discussion

The rat sarcoma (Ras) oncoprotein superfamily of small GTPases is involved in membrane-trafficking events (15). In particular, it has been indicated that Rab25, which is specific to epithelial cells, is involved in human cancers (11,16). Previous studies have suggested that Rab25 can increase the aggressiveness of human cancer and promote the progression of the disease (12,17–19). By contrast, certain previous studies have reported that the downregulation of Rab25 expression can cause a more aggressive phenotype in colon cancer, triple-negative breast cancer and esophageal squamous cell carcinoma (9,10,20,21).

To date, there have been limited reports on the importance of Rab25 in ccRCC (22). The discrepancies in the available data led us to investigate the Rab25 expression pattern in human ccRCC and its clinical significance. In this study, higher levels of Rab25 protein were identified by western blot analysis in ccRCC tissue samples than in paired adjacent noncancerous tissues. In addition, the immunohistochemistry data showed that Rab25 protein is highly expressed in ccRCC tissues. Although we did not investigate the potential mechanisms by which Rab25 is involved in metastasis, a previous study suggested that it may be by promoting the invasive migration of cells, by localizing and maintaining integrin α-V/β-1 expression at the tips of extending pseudopodia, and/or by modulating cellular processes, including the proliferation, survival and migration of epithelial tumor cells, in order to increase the rates of cancer progression and its aggressiveness (17). Since the role of Rab25 as an anti-apoptotic protein has been associated with breast cancer progression and cell survival, the overexpression of Rab25 may be essential to the survival of these cell types. In addition, studies have suggested that the anti-apoptotic molecule heat shock protein 27 (HSP27) regulates breast cancer cell growth, and HSP27 and HSP70 are linked to metastasis in epithelial ovarian cancer (12,18,19). Tumor invasion and metastasis are known to be responsible for the majority of cancer-related mortalities. In the present study, the high expression of Rab25 protein was significantly correlated with the primary tumor stage, lymph node metastasis and distant metastasis, suggesting that Rab25 overexpression contributes to the invasion and metastasis of ccRCC.

Additionally, the association between Rab25 protein expression and prognosis in ccRCC was examined. Rab25 expression was significantly associated with poor prognosis in patients with ccRCC. The Cox proportional-hazards model analysis showed that Rab25 expression was an independent prognostic factor for overall survival time. These findings suggest that Rab25 expression may be involved in the progression of ccRCC. Further investigations are required to clarify the mechanisms of Rab25 on outcome in ccRCC.

In conclusion, the present study showed that elevated expression of Rab25 raised the metastatic potential of ccRCC cells. Furthermore, Rab25 was an independent prognostic factor for patient survival time and was closely associated with lymph node metastasis. Targeting Rab25 could be a novel strategy for the prevention of ccRCC metastasis. Further evaluation of Rab25 in ccRCC and non-clear cell RCCs, and with a larger scale of ccRCC samples, is warranted.

## Figures and Tables

**Table I:** Associations between Rab25 expression and clinicopathological variables in 107 patients with clear cell renal cell carcinoma.

		Rab25 staining		
			
Variable	Total (n)	Positive (n)	Negative (n)	P-value
Gender				0.125
Male	79	55	24	
Female	28	15	13	
Age (years)^a^				0.082
<56	44	33	11	
≥56	63	37	26	
T stage				<0.001
T_1,2_	74	58	16	
T_3,4_	33	12	21	
N stage				<0.001
N_0_	93	68	25	
N_1,2_	14	2	12	
M stage				<0.001
M_0_	96	69	27	
M_1_	11	1	10	
TNM stage				<0.001
I–II	66	58	8	
III–IV	41	12	29	
Histological grade				0.004
G_1,2_	61	47	14	
G_3,4_	46	23	23	
Histology type				0.022
Clear cell	86	51	35	
Papillary	14	12	2	
Chromophobe	7	7	0	

a The median age is 56 years.

T, tumor; N, node; M, metastasis; G, grade.

**Table II:** Univariate and multivariate analyses of overall survival rate in 107 patients with clear cell renal cell carcinoma.

	Univariate			Multivariate		
		
Risk factors	HR	95% CI	P-value	HR	95% CI	P-value
ECOG PS	5.43	2.78–10.58	<0.001	2.45	1.19–5.05	0.020
Tumor size	4.46	1.73–11.48	0.002	1.24	0.42–3.61	0.700
Fuhrman’s grade	10.57	5.07–22.03	<0.001	3.96	1.63–9.64	0.002
VI	9.46	4.27–20.94	<0.001	4.63	1.80–11.92	0.001
Rab25 expression	7.20	2.77–18.73	<0.001	3.43	1.13–10.38	0.023

CI, confidence interval; HR, hazard ratio; ECOG PS, Eastern Cooperative Oncology Group performance status; VI, vascular invasion.
